# P Wave Dispersion is Increased in Pulmonary Stenosis

**Published:** 2006-01-01

**Authors:** Namik Ozmen, Bekir Sitki Cebeci, Ejder Kardesoglu, Turgay Celik, Mehmet Dincturk, Ergun Demiralp

**Affiliations:** * Assistant Professor; † Associate Professor; ‡ Professor; Gulhane Military Training Hospital, Cardiology service, Istanbul, Turkey

**Keywords:** P wave dispersion, pulmonary stenosis

## Abstract

**Aim:**

The right atrium pressure load is increased in pulmonary stenosis (PS) that is a congenital anomaly and this changes the electrophysiological characteristics of the atria. However, there is not enough data on the issue of P wave dispersion (PWD) in PS.

**Methods:**

Forty- two patients diagnosed as having valvular PS with echocardiography  and 33 completely healthy individuals as the control group were included in the study. P wave duration, p wave maximum (p max) and p minimum (p min) were calculated from resting electrocariography (ECG) obtained at the rate of 50 mm/sec. P wave dispersion was derived by subtracting p min from p max. The mean pressure gradient (MPG) at the pulmonary valve, structure of the valve and diameters of the right and left atria were measured with echocardiography.  The data from two groups were compared with the Mann-Whitney U test and correlation analysis was performed with the Pearson correlation technique.

**Results:**

There wasn’t any statistically significance in the comparison of age, left atrial diameter and p min between two groups.  While the MPG at the pulmonary valve was 43.11 ± 18.8 mmHg in PS patients, it was 8.4 ± 4.5 mmHg in the control group. While p max was 107.1 ± 11.5 in PS group, it was 98.2 ± 5.1 in control group (p=0.01), PWD was 40.4 ± 1.2 in PS group, and 27.2 ± 9.3 in the control group (p=0.01)Moreover, while the diameter of the right atrium in PS group was greater than that of the control group, (38.7 ± 3.9 vs 30.2 ± 2.5, p=0.02). We detected a correlation between PWD and pressure gradient in regression analysis.

**Conclusion:**

P wave dispersion and p max are increased in PS. While PWD was correlated with the pressure gradient that is the degree of narrowing, it was not correlated with the diameters of the right and left atria.

## Introduction

P wave dispersion (PWD) indicates the spreading of the sinus stimulus in the atria. geneity in the depolarisation of the atria and thus causes atrial fibrillation. Stretching of the atria due to pressure and/or volume load, electrolyte imbalance or increase in sympathetic activity are the main causes that increase PWD.  It is recognised that PWD and hence the probability of atrial fibrillation are increased in pathological conditions that increase the left atrial pressure such as mitral stenosis, aortic stenosis and systemic hypertension [1-3] or in pathological conditions that increase the right atrial pressure such as atrial septal defect [4,5] and COPD (Chronic Obstructive Pulmonary Disease) [6]. On the other hand, chronic renal insufficiency accompanied by intravascular volume load and electrolyte imbalance also increase PWD [7].

In atrial septal defect or COPD, the right atrial pressure increases, and the right atrium stretches and becomes more enlarged. Thus, the depolarisation duration of the right atrium is prolonged and this causes an increase in PWD. Valvular pulmonary stenosis (PS) results from the fusion of pulmonary cusps in the intrauterine period and constitutes 7% of congenital heart diseases. Pulmonary stenosis which is a relatively rare congenital heart disease causes concentric hyperthrophy of the right ventricle, increase in end-diastolic pressure and increase in right atrial pressure.  This condition can be expected to lead an inrease in PWD. P wave dispersion in PS patients was not defined previously. In this study, we  have investigated PWD in patients with valvular PS.

## Material and Methods

A total of 42 patients with valvular PS diagnosed by echocardiography performed after cardiac examination were admitted into the study. 33 apparently healthy age-matched subjects were included to the study as the control group. Healthy status was documented using physical examination, ECG, chest X-ray and echocardiography. Patients with any acquired or congenital heart disease, valvular disease, pericardial disease, bronchial asthma, COPD or any systemic disease were excluded from the study. All patients provided written consent and the study was  approved by the local Ethics Committee.

### Echocardiography

The left and right ventricles and their valvular structures were assessed in detail using the standart left lateral decubitus position Vingmed system FiVe with 2.5 MHz probe (GE, Holten, Norway). In the apical four chamber image, the diameter of the right atrium (RAD) and the diameter of the left atrium (LAD) were measured at the level of the annulus of mitral and tricuspid valves in millimeters (mm), respectively defined as the distance from the lateral wall of the right atrium to the interatrial septum, and from the lateral wall of the left atrium to the interatrial septum. The pulmonary valve was structurally assessed in the parasternal short axis image. Then, the mean and peak pressure gradients over the pulmonary valve were obtained using CW Doppler. Valvular pulmonary stenosis was defined as a dysplastic or dismorphic pulmonary valve with a peak systolic pressure gradient (PG) more than 20 mm Hg and a mean pressure gradient more than 10 mm Hg.

### Electrocardiography (ECG)

Twelve-lead surface ECG was obtained from all patients and controls at a paper of 50 mm /s with 1 mV/cm   standardization after resting 5 minutes.  Subjects were allowed to breathe freely but not to speak or cough during recording.  The P wave duration was manually measured by a cardiologist blind to the study, using a magnifying lens. The onset of the P wave was defined as the junction between isoelectric line at the beginning of the P wave deflection and the offset of the P wave and the isoelectric line. The leads with the onset or offset of the P wave not clearly determined were excluded from the analysis. 2 patients whose ECGs not clear enough were also excluded from the study. This method was used in previous studies by different investigators [8,9]. P maximum (p max) duration and P minimum (p min) duration were measured from 12-lead ECG. Then,  PWD was defined as the difference between p max and  p min.

### Statistical assessment

The data from the patient and control groups were compared with each other using SPSS 11.0 statistical software package (SPSS Inc., Chicago, IIinois, USA) program. PWD, Pressure gradient, right and left atrium diameters were compared with the Mann-Whitney U test, and the correlation between PWD and the other variables was seeked using the Pearson correlation co-efficient. The data were expressed as mean ± SD. P values under 0.05 were considered  statistically significant.

## Results

Forty two patients with valvar PS were included in the study; 35 were males, 7 were females  and the mean age was 22.1 ± 4.2 years. The control group was comprised of 33 subjects; 23 were men, 10 were women and the mean age was 23.8 ± 3.1 years. In the PS group, only 5 patients complained of  palpitations, but rare atrial premature beats were detected,  and atrial fibrillation was not observed in any patient. There was not any statistical significance between groups as to the mean ages or heart rate (82.4 ± 8.5 vs 78.2 ± 7.2, p>0.05).

 While the mean pressure gradient at the pulmonary valve was 43.1 ± 18.8   mmHg in the PS group, it was 8.4 ± 4.5 mmHg in the control group (p=0.01). The average right atrium diameter was 38.7 ± 3.9 mm in PS group while it was 30.2 ± 2.5 mm in control group (p=0.02). However, the average left atrium diameter was 32.2 ± 4.1 mm in PS group and 30.2 ± 2.8 mm in control group, and there was no significant difference (p=0.64). According to this, while the right atrium was significantly larger in PS patients, the left atrium diameter was not different from that of the control group (Table 1).

While p max was 107.1 ± 11.5 ms in patients with PS, it was 98.2 ± 5.1 ms in the contol group (p=0.01), and while PWD was 40.4 ± 1.2 ms in PS group, it was 27.2 ± 9.3 ms in the control group (p=0.01). Both p max and PWD values were significantly greater in the PS group. However, p min was similar in both groups (66.1±1.2 ms vs 71.2 ±4.3 msn p>0.05) (Table 2).

In the pulmonary stenosis group, PWD, p max and mean pressure gradient and the right atrium diameter were significantly greater than control group. A correlation analysis of PWD with pressure gradient and right and left atria diameters was performed; while PWD was correlated with pressure gradient (r = 0.38, p = 0.009), there was no correlation between PWD and the right and left atrium diameters ( r = - 0.05, p= 0.74 and r=0.15, p= 0.30, respectively).

## Discussion

The main findings of our study are as follows: 1. PWD is increased in pulmonary stenosis. 2. PWD shows correlation with the pressure gradient. 3. PWD is not greatly affected by the right atrial diameter in PS patients. The low AF incidence in PS patients may be attributed to the intervention in this patient group before PWD is further increased.

Increased PWD is an electrocardiographic marker that has been associated with the inhomogeneous and discontinuous propagation of sinus impulses. It can be defined as the difference between maximum and minimum p-wave duration. Prolongation of intra-atrial and interatrial conduction time and inhomogenous prolongation of sinus impulses are known electrophsiologic characteristics of atria prone to fibrillation [8,10]. Moreover, the correlation between the presence of intra-atrial conduction abnormalities and the induction of paroxysmal atrial fibrillation have been documented [11,12]. Also, the increase in the pressure or volume load of the atria, stretching of the atria, electrolyte imbalance and increase in sympathetic activity cause PWD to increase. Stretching and enlargement of the atria, disorganisation of the atrial muscles even if partial, and fibrosis in the atrial wall lead to the prolongation of atrial conductance and impairment of its homogeneity.

It is known that atrial conduction is impaired and PWD is prolonged in haemodialysis patients as a result of intravascular volume load and electrolyte imbalance [7].  Reduction of intravascular volume with diuretic use, on the other hand, decreases the duration of p max and PWD [13]. However, it is evident that not volume load but pressure load is involved in PS.

On the other hand, PWD and the duration of p max are increased also in aortic stenosis and mitral stenosis in which the pressure load in the left atrium is increased. Turhan et al [2], showed that p max and PWD were higher than in healthy subjects. However, they reported that there was no difference with respect to echocardiographical parameters. In another study by Turhan et al. again, they showed that in mitral stenosis, a prominent reduction occurred  in p max, PWD, the diameter of the left atrium and the transvalvular pressure gradient with mitral baloon valvuloplasty  24 hours after and one month after the procedure [1]

It is observed that p max is prolonged in both the mentioned studies and our study. This is interpreted as an indicator of interatrial conduction impairment independent of the atrium diameter [14]. Likewise, Dilaveris [10] and Ishimoto et al. [15] reported that there was no correlation between p max and PWD and the left atrium diameter. In our study, although the right atrium diameter was increased, we showed that PWD was not correlated with the right or left atrium diameter but correlated with the pulmonary valve pressure gradient. As in the previous studies, p min did not differ between the two groups in our study either.

Guray et al. showed  that p max and PWD were prolonged in patients with ASD [4]. In that study while PWD was correlated with Qp/Qs, the left atrium diameter was the same with the control group. However, the right atrium diameter was not mentioned in that study. In our study, the right atrium diameter was significantly greater than in the control group.

The only study related to the right atrium and right cardiac pressure loading is the study by Tukek et al. [6] on PWD in patients with COPD. In this study, the researchers demonstrated that PWD, p max and p min values were greater in patients with COPD compared with the values in the control group and that in the same fashion PWD was greater in patients with paroxysmal AF compared to patients without paroxysmal AF. They also stated that there was no relationship between PWD and pulmonary functions, blood gases and right and left atrium functions. Our study is similar to this study in that it investigates the right atrium pressure load and PWD. However, our patients were from a younger age group and consisted of individuals that did not have COPD or any other systemic disease.

Morover, PS is relatively rare congenital heart disease, there is not enough information on PWD in PS. That these patients are detected and treated at a young age may be the reason for this lack of information. Considered from this perspective, our study might be interesting also because it is the first of its kind.

## Conclusion

P wave dispersion and p max are increased in PS which is a congenital heart disease. There is a correlation between the pressure gradient that is the degree of narrowing and PWD. On the other hand, there is no correlation with the right and left atrium diameters. However, we think that studies involving larger patient populations are warranted.

## Figures and Tables

**Table 1:**
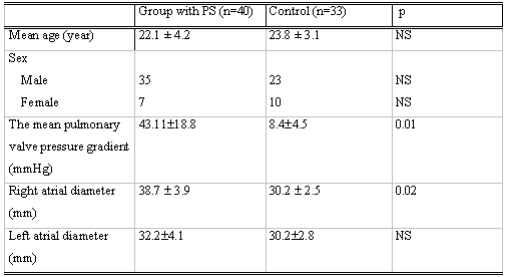
The clinical signs of the study population

**Table 2:**
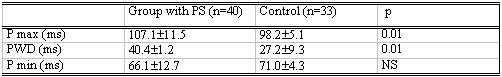
PWD, P max and P min values of the study population
